# Prospective stratification of patients at risk for emergency department revisit: resource utilization and population management strategy implications

**DOI:** 10.1186/s12873-016-0074-5

**Published:** 2016-02-03

**Authors:** Bo Jin, Yifan Zhao, Shiying Hao, Andrew Young Shin, Yue Wang, Chunqing Zhu, Zhongkai Hu, Changlin Fu, Jun Ji, Yong Wang, Yingzhen Zhao, Yunliang Jiang, Dorothy Dai, Devore S. Culver, Shaun T. Alfreds, Todd Rogow, Frank Stearns, Karl G. Sylvester, Eric Widen, Xuefeng B. Ling

**Affiliations:** 1 HBISolutions Inc., Palo Alto, CA 94301 USA; 2 Departments of Pediatrics, Stanford University, Stanford, CA 94305 USA; 3 Department of Surgery, Stanford University, S370 Grant Building, 300 Pasteur Drive, Stanford, CA 94305 USA; 4 Statistics Stanford University, Stanford, CA 94305 USA; 5 HealthInfoNet, Portland, ME 04103 USA; 6 Academy of Mathematics and Systems Science, Chinese Academy of Sciences, Beijing, 10019 China

**Keywords:** ED revisit prediction, Prospective validation, Statistical modeling, EMR

## Abstract

**Background:**

Estimating patient risk of future emergency department (ED) revisits can guide the allocation of resources, e.g. local primary care and/or specialty, to better manage ED high utilization patient populations and thereby improve patient life qualities.

**Methods:**

We set to develop and validate a method to estimate patient ED revisit risk in the subsequent 6 months from an ED discharge date. An ensemble decision-tree-based model with Electronic Medical Record (EMR) encounter data from HealthInfoNet (HIN), Maine’s Health Information Exchange (HIE), was developed and validated, assessing patient risk for a subsequent 6 month return ED visit based on the ED encounter-associated demographic and EMR clinical history data. A retrospective cohort of 293,461 ED encounters that occurred between January 1, 2012 and December 31, 2012, was assembled with the associated patients’ 1-year clinical histories before the ED discharge date, for model training and calibration purposes. To validate, a prospective cohort of 193,886 ED encounters that occurred between January 1, 2013 and June 30, 2013 was constructed.

**Results:**

Statistical learning that was utilized to construct the prediction model identified 152 variables that included the following data domains: demographics groups (12), different encounter history (104), care facilities (12), primary and secondary diagnoses (10), primary and secondary procedures (2), chronic disease condition (1), laboratory test results (2), and outpatient prescription medications (9). The *c*-statistics for the retrospective and prospective cohorts were 0.742 and 0.730 respectively. Total medical expense and ED utilization by risk score 6 months after the discharge were analyzed. Cluster analysis identified discrete subpopulations of high-risk patients with distinctive resource utilization patterns, suggesting the need for diversified care management strategies.

**Conclusions:**

Integration of our method into the HIN secure statewide data system in real time prospectively validated its performance. It promises to provide increased opportunity for high ED utilization identification, and optimized resource and population management.

**Electronic supplementary material:**

The online version of this article (doi:10.1186/s12873-016-0074-5) contains supplementary material, which is available to authorized users.

## Background

### Background and importance

The utilization of emergency department (ED) services in the United States (U.S.) is growing at an alarming rate [1]. Between 1999 and 2007, the annual number of U.S. ED visits grew at roughly twice the rate of population growth [2]. When patients return to the ED after discharge, it is generally believed that revisits are attributable to the nature of the disease, medical errors, and inadequacy of initial evaluation or treatment [3]. ED revisits can involve patients who are in a high-risk population of specific demographics [4]. However, the circumstances surrounding the ED revisit are poorly understood. Some ED-discharged patients return for non-emergency problems [3], while others could be underserved due to the lack of local primary care and/or specialty availability, which significantly increases overall emergency use [5]. Recent evidence from U.S. Oregon’s health insurance experiment found that a limited expansion of a Medicaid program for uninsured, low-income adults increased ED use [6].

Improving appropriate utilization of emergency services is an important strategy for improving health outcomes and controlling healthcare expenditures [7]. Presuming a large proportion of ED return visits are preventable, studying the quality assurance of ED care becomes a necessary task to improve and maintain service at a high level.

Greater utilization of advanced analytic computing methods on patient clinical histories has led to the development of several algorithms to assess patient risk. Early efforts have included risk prediction models for hospital readmission [8, 9] and repeat ED visit for patients with distinct demographic features [10–14].

Unscheduled ED revisits may occur for any reason and can be separated by days, weeks, months or years. Short term ED revisits, 3 to 7 days, could be due to the received poor quality, possible errors, or adverse events. 6-month ED return, which can be caused either by medical errors, mismanagement, or unexpected reasons, tended to raise healthcare utilization issues. Risk assessment tools for 6-month ED return can allow high-risk patient identification who might require personalized local care and/or specialty, and some targeted interventions. 30-day ED return also tended to raise healthcare utilization issues. An investigation in 2011 from 13 U.S. states showed that there were only 7 % patients having 1-week revisit, 10 % having 2-week revisit, and more than 25 % having 30-day revisit [15]. In our database there were greater than 40 % of patients in Maine State who revisited ED within 30 days, clearly imposing a burden on hospital resource utilization. Although such models demonstrate utility in limited settings [16], patient risk prediction remains a poorly understood and complex endeavor. It is more challenging to predict 30-day return, and LaMantia MA et al. [9] failed to produce models predicting 30-day ED return accurately for the elderly. Currently used patient risk-prediction models rely on retrospective administrative data [8] that are disproportionately influenced by the high rate of previous ED admissions that do not necessarily correlate with ongoing risk for future ED admission [17]. Most risk assessment studies focus on patients within specific payer groups, e.g. Medicare / Medicaid, within specific age, and/or within specific disease groups [12, 18–21].

With the increased adoption of electronic medical record (EMR) systems and the development of health information exchanges (HIE) in the U.S., healthcare organizations have better and more comprehensive access to patients’ comprehensive medical histories. We have successfully developed and prospectively tested a risk assessment tool of 30-day ED return across statewide population within Maine HIE [22]. It was derived through statistical learning from a high-dimensional, longitudinal EMR data source containing demographics and prior-year clinical histories. An overall model c-statistic of 0.72 was achieved. The tool was successfully integrated into the statewide HIE services to compute patients’ daily risk updates. Success of this 30-day risk assessment tool highlighted the opportunity of predicting future health resource utilization based on the past EMR information, driving us to develop a 6-month ED return risk assessment tool via the same approach.

In this study, we set to apply the statistical learning from patient data contained in a statewide HIE of longitudinal patterns to identify risk factors that strongly influence the probability of a future 6-month ED revisit. This effort has been part of a collaborative project with HealthInfoNet (HIN), a nonprofit organization operating Maine’s HIE. HIN operates a centralized model HIE, that in near real-time connects, aggregates and centrally stores data from thirty-four Maine hospitals and physician practices as well as clinics that offer health care services to over one million patients. HIN data is from all payers, all ages, and all diseases.

### Goals of this investigation

Most prior studies on ED returns were focused on quality improvements to identify possible errors or mismanagement that occurred on the past ED visit, therefore, timeframe for return visits has generally been short (3 to 7 days) [23–25]. In the State of Maine, greater than 70 % ED patients with no past ED history and 80 % with past ED history revisited ED within 6 months past the index visit. Accurate identification of patient populations at risk for ED return visits is a critical component for targeting post discharge interventions to high-risk patients in an effort to improve the healthcare resource allocation. We set to identify patients of high ED utilization, who might be better managed with personalized local care and/or specialty, and who might require targeted interventions. To the best of our knowledge, this prospective study is the first to identify high utilization patterns of statewide ED patients across all payers, all diseases and all age groups.

## Methods

### Ethics statements

This work was done under a business/product development arrangement between HIN and HBI Solutions, Inc. and the data use is governed by the HIPAA business agreement (BAA) between HIN and HBI. No PHI was released for the purpose of research. Instead, HBI completed the product development that was the foundation for our agreement and then reported on the findings resulting from applying this model to the products/services that HIN is now deploying in the field. Because this study analyzed deidentified data, the Stanford University Institutional Review Board considered it exempt (October 16, 2014).

### Study design, setting, and selection of participants: overview of study design

**Fig. 1:**
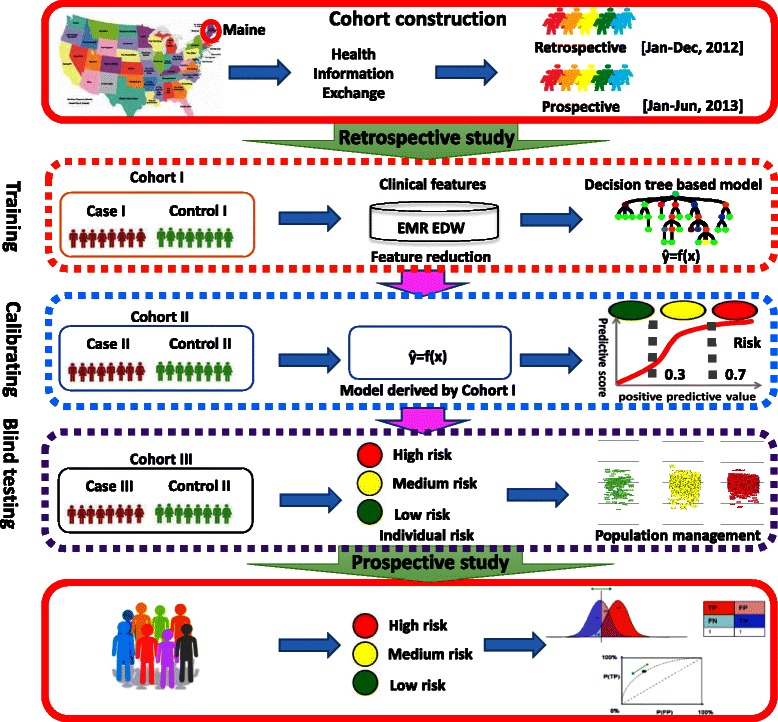
Study design to develop the ED revisit predictive algorithm. A flow chart of 5 steps from cohort construction to prospective validation is demonstrated. Maine healthcare information was extracted to build a retrospective and prospective cohorts. Samples in the retrospective cohort were randomly split into 3 sub-cohorts (Cohort I, II and III) for training, calibrating and blind testing of a decision_tree_based predictive model. Two thresholds of 0.3 and 0.7 were applied to the ranked outputs of the model to divide the population into low, medium and high risk groups. The model together with the risk stratification was validated on the prospective cohort by PPV, sensitivities and ROC

The statistical learning to forecast future 6-month ED revisit risk consisted of two phases: retrospective modeling and prospective validation (Fig. 1). It was a primary analysis of prospectively collected EMR data including administrative data and other medical history data.

### Population

The study intended to cover post discharge ED revisits across all payers, all diseases and all age groups. Patients visiting any HIN connected facility from January 1, 2012 through December 31, 2013, were eligible for study. Patients that died, as identified through an encounter disposition code, were excluded during the study time frame of 2012 and 2013. ED visits that transferred from another ED were excluded as these were treated as one ED visit, and not multiple.

### Data warehouse

We constructed an enterprise data warehouse consisting of all Maine’s HIE aggregated patient histories. The details of data extraction, management and storage were described in the Additional file 1. Incorporated data elements from EMR encounters include patient demographic information (including age, gender and social status), encounter-based laboratory and radiographic tests (i.e. lab or radiographic tests performed in a ED encounter) coded according to Logical Observation Identifier Names and Codes (LOINC), patient-based past-12-month outpatient medication prescriptions coded according to National Drug Code (NDC), encounter-based primary and secondary diagnosis and procedures which are coded according to the *International Classification of Diseases, 9*^*th*^*Revision, Clinical Modification* (ICD-9-CM). Census data from the U.S. Department of Commerce Census Bureau were integrated into our data warehouse. Therefore, in addition to the HIN features, we categorized patients by socioeconomic status utilizing residence zip codes as an approximation to the average household mean and median family income and average degree of educational attainment. Although Maine HIE patient clinical histories were described by a large number of features (totally 14,860) for each subject, a high proportion of them were highly sparse with rate of more than 99 % missing data, like some chronic diseases, medications, diagnosis and procedures. Such characteristics made a considerable number of features to have less power of prediction, which needed to be removed from the model inputs before the training process. To efficiently eliminate these features, we did data variance analysis to identify and remove those with least variances in each feature category [26]. In practical health information system or EMR system, most of the data elements are highly sparse, and are not commonly associated with repeated ED visits. Highly sparse features contain little information, introduce unnecessary noise, and are of little power of prediction. Therefore, we screened the features by applying low-variance criteria to the training population (293,461 observations and total 14,860 features). After this preprocessing, features with more than 99.9 % missing data were eliminated. As a result, a set of patient clinical historical features in the prior 12 months to the ED discharge date was compiled (see Additional file 2). One of the key features was whether the patient had a chronic medical condition. This feature was defined using the AHRQ Chronic Condition Indicator [27] (CCI) which provides an effective way to categorize ICD-9-CM diagnosis codes into one of two categories: chronic and non-chronic.

### Cohort construction

**Fig. 2:**
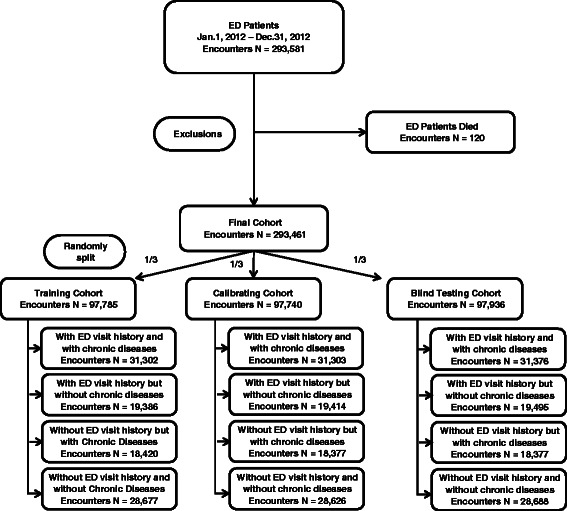
Retrospective cohort construction. The final cohort includes 293,461 ED encounters between Jan 1, 2012 and Dec 31, 2012. 120 encounters associated with patients who have died were excluded from the database. It consisted of 4 subgroups based on the past-year ED visit and chronic disease histories, and was randomly split into 3 parts for training, calibrating and blind testing purposes

**Fig. 3:**
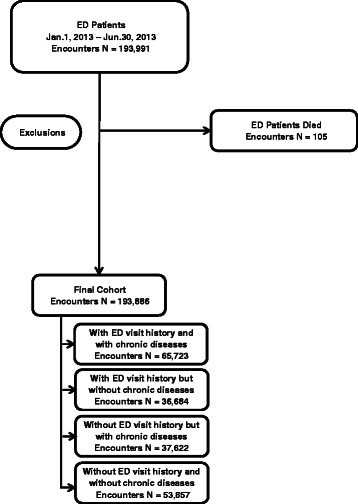
Prospective cohort construction. The final cohort includes 193,886 ED encounters between Jan 1, 2013 and Jun 30, 2013. 105 encounters associated with patients who have died were excluded from the database

To develop the model, a retrospective cohort of 293,461 ED encounters (Fig. 2), between January 1, 2012 and December 31, 2012, was assembled. To validate, a prospective cohort of 193,886 encounters (Fig. 3) between January 1, 2013 and June 30, 2013 was constructed. Both cohorts associated patients had similar demographics and one-year comprehensive clinical histories before the discharged date that enabled a determination of subsequent post discharge ED revisit risk (see Additional file 3).

### Model development – a retrospective analysis

In the present study an ED revisit prediction algorithm was developed with a statewide post discharge 6-month ED revisit risk measure. The measure comprised a single summary score, derived from the results of a “forest” of the most discriminative decision trees upon 1 year of the encounter history. The measure calculated each ED subject’s probability of a future 6-month ED revisit at the ED discharge, and mapped it to a risk score ranging between 0 and 100, where subjects having scores less than 30, between 30 and 70, and higher than 70 were recognized as the low, medium and high risk groups, respectively.

The retrospective cohort case (post discharge 6-month ED revisit counts > 0) and control (post discharge 6-month ED revisit counts = 0) samples were randomly partitioned into three groups (I, II, III, Fig. 1) for model training, calibration and blind testing purposes. An ensemble decision-tree-based model was developed using the prior year clinical history data [28]. The detailed modeling process was demonstrated in the Additional file 4.

### Feature selection

As mentioned in “Goals of this investigation” section, in our implementation, one of the objectives was to select the least number of representative features predictive of future 6-month ED revisit risk and achieve optimal case finding sensitivity while maintaining the targeted positive predictive value (PPV > 70 %) based on selected features. A flow chart of the feature selection process is shown in Additional file 5. Features having top variances and top weights of the derived random forest model were selected, and then a sensitivity analysis was performed with different feature variable numbers in order to identify the least variable number giving the maximum sensitivity as well as high PPV which constructed the final predictive model. Our statistical learning identified 152 variables predictive of future defined 6-month risk of ED visit: demographics groups (12), history of different encounters (104), facilities (12), history of primary and secondary diagnoses (10), history of primary and secondary procedures (2), comorbidities (1), laboratory test results (2), and history of outpatient prescription medications (9). These features’ shrunken difference [29] (Prospective analysis: see Additional file 6) were grouped according to the categories identified above. These discriminant features’ absolute values of the shrunken differences, among the low, medium, and high risk outcomes, differed more than the case (with future ED) and control (without future ED) outcomes, prospectively demonstrating the effectiveness of these features in the risk stratification.

### Model validation – a prospective analysis

The clinical application of the 6-month post discharge ED revisit risk measure was deployed for prospective validation on the HIE data in Maine. Patients discharged from the ED were prospectively profiled to calculate future 6-month ED revisit risk measures using the clinical applications deployed at HIN. The receiver operating characteristics (ROC) [30] and time to event analyses were performed to gauge the model performance and effectiveness of the risk stratification.

### Clinical pattern identification of patients associated with high-risk ED revisits

Principal component analysis was utilized to identify clinically relevant groups of patients of high risk for post discharge 6-month ED revisit with similar patterns of demographics, primary diagnosis and procedure, and chronic disease conditions. Clustering patterns between retrospective and prospective cohorts were compared to further validate the validity of the high-risk case finding algorithm. The details of the clustering procedure are shown in Additional file 7.

## Results

### Characteristics of study subjects

In addition to clinical and field care-giver judgments, we reviewed a “time to event” curve of ED revisits of the retrospective cohort, to determine whether 6-month post discharge ED revisit assessment is clinically reasonable in that a large proportion of patients had ED returns with 6 months that accounts for considerable resource demands. The ED revisit “time-to-event curve” (see Additional file 8) showed a pattern of rapid accrual with a stable and consistent ED revisit rate thereafter. The ED revisit curve reduced to less than 20 % within 6 months from the discharge time, indicating that a 6-month cutoff was reasonable and appropriate for this study. Patients in the retrospective and prospective cohorts were also similar in incidence of future 6-month ED visits (retrospective: 43.0 %; prospective: 44.8 %; see Additional file 3). Our exploratory analysis (see Additional file 9) of the retrospective cohort showed that the percentage of ED encounters with future 6-month revisits increased as a function of either historic ED visit counts or the presence of chronic disease diagnoses, therefore, these two features were strongly associated with patients’ risk for post discharge 6-month ED revisits.

### Main results

The ED revisit algorithm produces a risk score (from 0 to 100, as a continuous variable) for each subject at the time of ED discharge to assess the risk of ED revisit. In regard to the threshold parameter for subgrouping patients of different ED return risks, low (score < 30), intermediate (score ≥ 30 and score < 70), high (score ≥ 70), it was chosen arbitrarily. However, in the dashboard tool we developed and deployed at Maine HIE, the field users can choose any threshold setting to construct cohort of different risks for targeted patient care. The model performance was tabulated in Table 1 with thresholds of 50, 70, and 80. At a risk score threshold of 50, the algorithm identified 75.8 % (retrospective analysis) and 71.6 % (prospective analysis) of patients that returned to ED after 6 months; as well as PPVs are 61.4 % (retrospective analysis) and 59.7 % (prospective analysis) (Table 1). At risk score threshold levels of 70 and 80, PPVs increased to 69.6 and 83.0 % in retrospective analysis, and 66.9 and 79.4 % in prospective analysis respectively. At the 70 and 80 thresholds, the algorithm still found an impressive percentage of ED revisits wherein sensitivities decreased to 59.8 and 38.9 % in retrospective and 54.4 and 32.4 % in prospective analysis respectively. The receiver operating characteristic curve analyses showed that there was a 74.2 % (retrospective) or 73.0 % (prospective) probability that a randomly selected ED discharged patient with a 6-month post discharge ED revisit will receive a higher risk score than a randomly selected patient who will not have a future 6-month ED revisit.

**Table 1:** ED 6 month revisit risk stratification results

Characteristics	Retrospective			Prospective		
	(Jan. 1, 2012 – Dec. 31, 2012)			(Jan. 1, 2013 – Jun. 30, 2013)		
	Risk score threshold			Risk score threshold		
	50	70	80	50	70	80
No. of ED encounters	95,785	75,593	49,109	62,189	47,235	28,166
Positive predictive value	0.614	0.696	0.83	0.597	0.669	0.794
Sensitivity	0.758	0.598	0.389	0.716	0.544	0.324
Specificity	0.639	0.802	0.94	0.608	0.781	0.931
Average ED visits in the future 6 months	2.13	2.74	4.11	2.32	3.01	4.77

In developing the algorithm, we aimed to help potential care providers to assess the “opportunity case” (high-cost, high degree of utilization of services, multiple chronic conditions) for various risk scores and for different assumptions about the impact of the ED post discharge intervention. A “time to event” analysis (see Additional file 10) demonstrated that the ED revisit algorithm was capable of stratifying patients across a wide range of risk. Patients in higher risk categories returned to the ED earlier (prospective time to event analysis: *p* < 0.001) and more frequently (Table 1) over the post discharge 6-month period.

**Fig. 4:**
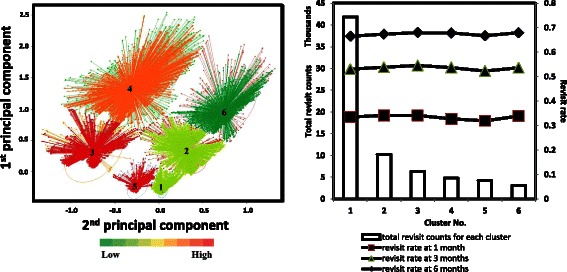
The ED predictive algorithm effectively risk-stratified the prospective patient cohort for future 6-month ED visit. *Left panel*: Unsupervised clustering of the high-risk patients identified distinct subgroups in the prospective cohort. Color-coding reflects the ED resource utilization of the high-risk patients in the next 6-month post discharge. *Right panel*: Prospective ED revisit rates and counts for each cluster at 1, 3 and 6 months’ points

To test the hypothesis that ED revisit high-risk patients (score ≥ 70) can be partitioned into subgroups with similar patterns of demographics, primary diagnosis and procedure, and chronic disease conditions to allow subsequent targeted care, patients at high risk for post discharge 6-month ED revisit underwent unsupervised cluster analysis. Our prospective analysis (Fig. 4, left panel) revealed a pattern of six distinct sub-groups among the high-risk patients, and these clinically relevant clusters (Table 2) grouped around multiple “anchoring” demographic and chronic disease conditions with different ED resource utilization patterns (Fig. 4, right panel). The largest cluster (#1) was characterized by predominantly young adult patients (between the ages of 19 and 34), and is the only group with 24.8 % patients without any chronic disease diagnosis history. Cluster #1 is the subgroup with the lowest consumption of average laboratory and radiology tests in the post ED discharge 6 months. In contrast, cluster #4 contained a relatively senior (35.2 % in age 50-65, 40.1 % in age > 65 age group) population with the highest number of average chronic disease diagnoses, and the highest average consumption of laboratory and radiology tests in the post ED discharge 6 months. Cluster #4 and #6 share similar patterns in (1) overall resource utilization in laboratory and radiology tests; (2) clinical history of approximately 0.25 % with cancer of pancreas diagnosis; (3) age group (Cluster #6, 77.6 % in the age > 65 group), however, cluster #6 consumed on average roughly half of the ED visits as did cluster #4 in the post ED discharge 6 months. Clusters #3, 5 shared similar age, gender profiles, and consumption profiles for laboratory and radiology tests, however, the two clusters displayed different disease diagnosis histories.

**Table 2:** Clustering of prospective ED-6-month high-risk patients according to demographics and the prior year clinical histories

	Characteristics	Cluster					
		1	2	3	4	5	6
	Number of encounters	41,887	10,218	6361	4798	4231	3101
Resource utilization	Average total lab test	124.75	230.98	278.51	514.73	175.55	351.06
	Average total Radiology	7.39	19.41	20.05	60	17.83	38.77
	Average future 6 month ED counts	2.68	2.56	4.53	4.06	4.58	2.15
Demographics	Sex (Female)	55.87	52.63	72.96	57.29	72.89	47.53
	Age new born	1.59	0.02	0.44	0	0.05	0
	Age 1–5	4.66	0.22	0.85	0	0.38	0
	Age 6–12	2.97	0.39	1.01	0	1.21	0
	Age 13–18	4.25	0.82	2.56	0.02	2.65	0
	Age 19–34	39.39	9.89	35.97	5.79	47.67	0.35
	Age 35–49	21.75	19.5	30.26	18.92	32.95	5.61
	Age 50–65	13.73	28.49	19.64	35.16	11.65	16.48
	Age >65	11.65	40.67	9.28	40.1	3.45	77.56
Chronic disease conditions	Total Chronic disease conditions	1.87	6.35	6.42	15.26	4.34	12.2
	Percentage of encounters without chronic diseases	24.8	0	0	0	0	0
	Other hematologic conditions	0.07	0.15	0.09	0.13	0.05	0.06
	Cancer of pancreas	0.03	0.14	0.02	0.25	0.07	0.23
	Pulmonary heart disease	0.07	0.03	0.09	0.06	0.05	0.03
	Transient cerebral ischemia	0.06	0.06	0.06	0	0.02	0
	Diabetes mellitus with complications	0.05	0.02	0.03	0	0	0.06
	Systemic lupus erythematous & connective tissue disorders	0.05	0.04	0.02	0	0.02	0
	Disorders of lipid metabolism	0.04	0.05	0	0	0.02	0.06
	Anxiety disorders	0.03	0.06	0.02	0.06	0	0
	Genitourinary symptoms and ill defined conditions	0.02	0.03	0.02	0.04	0	0.03
	Epilepsy convulsions	0.01	0.01	0.02	0.08	0	0.06
	Hyperplasia of prostate	0.03	0	0.02	0	0	0
	Immunity disorders	0.03	0	0.02	0	0	0
	Other complications of pregnancy	0.01	0.01	0.02	0.06	0.02	0.03
	Other diseases of bladder and urethra	0.01	0.01	0.02	0.06	0.02	0.03
	Cancer of kidney and renal pelvis	0.02	0.01	0.03	0.02	0.02	0
	Open wounds of extremities	0.02	0	0.02	0	0.02	0
	Other non epithelial cancer of skin	0	0.01	0.05	0	0.02	0.06
	Other nutritional endocrine and metabolic disorders	0	0.01	0.05	0	0.02	0.06
	Cancer other and unspecified primary	0.02	0	0	0	0.02	0
	Biliary tract disease	0.01	0.02	0	0	0	0.03

Characteristics of resource utilization, demographics and chronic disease conditions were summarized for each cluster. All the data shown within the headers of demographics and chronic disease conditions were expressed in percentages (%)

**Fig. 5:**
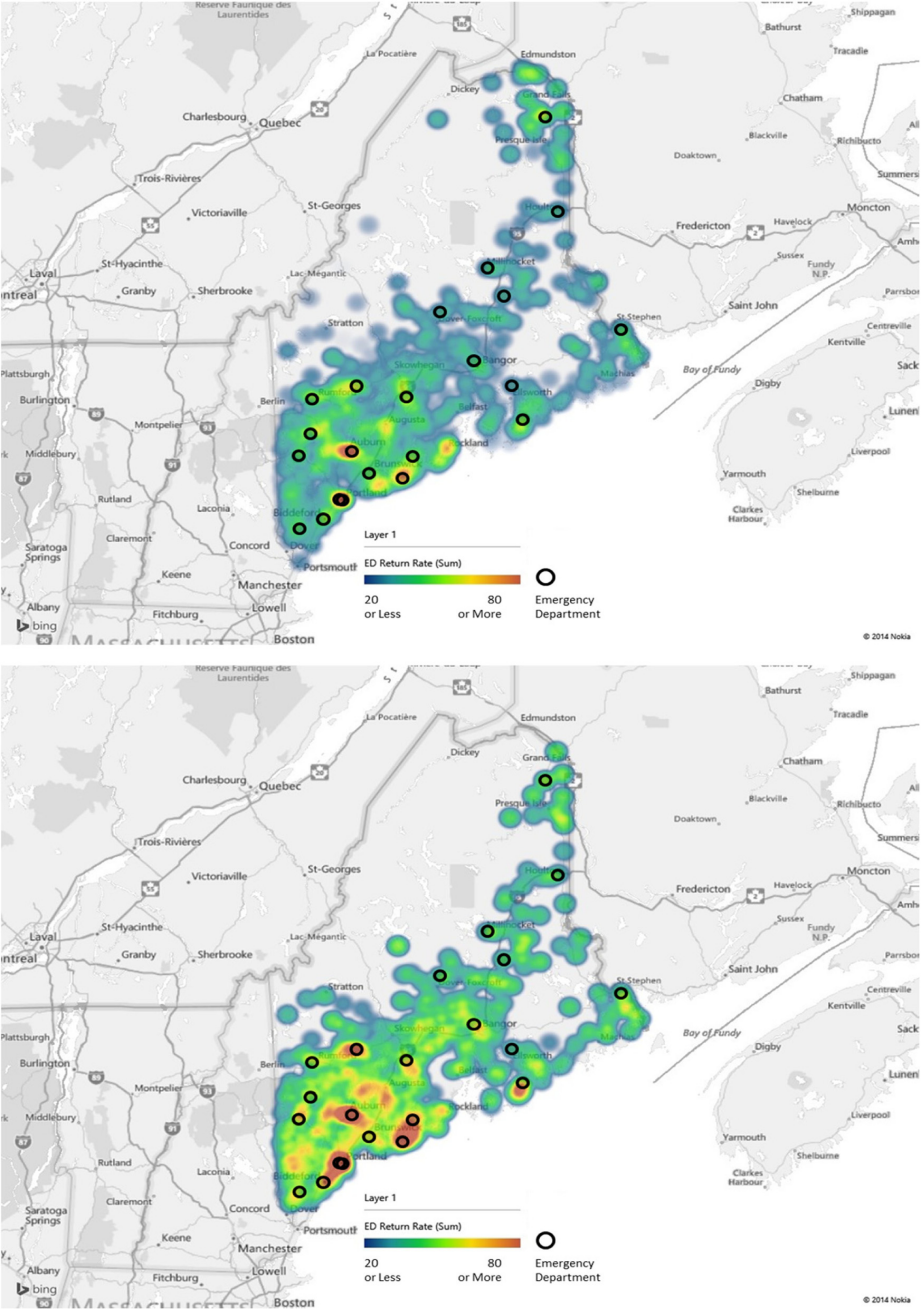
Geographic distribution of the prospective ED 6-month revisits and ED facilities in Maine State. Top panel: revisits of all ED encounters. Bottom panel: revisits of high risk ED encounters. The heatmap color metric indicates the average revisit rates in percentage (which equals to the number of ED encounters which returned within future 6 months divided by the total number of the ED encounters) at each location identified by zip codes. The maps were generated using Microsoft Power Map for Excel

A geographic distribution of future 6-month revisit rates of the prospective ED encounters was plotted as a heatmap with the geographic localization of ED facilities in Maine State (Fig. 5). Revisit rates were averaged for each zip code recorded at the first visit of each patient, and plotted on the map using different colors representing the rate values. It is clear that the high risk encounters demonstrating higher revisit rates across the state, which supports the risk stratification in our predictive algorithm. The map also shows high volume of ED revisits (around 80 %) was concentrated in the areas of Portland, Lisbon, Bristol, Rockland, Augusta and Southwest Harbor, while there were insufficient ED facilities in Rockland and Augusta.

## Discussions

We hypothesized that ED revisit risk can be forecasted from the statistical learning of ED discharged subjects’ comprehensive longitudinal clinical histories. Utilizing the population based HIE facilitated the development and prospective testing of the ED revisit risk stratification algorithm presented here whereby each ED discharge triggered an analysis of subsequent revisit risk. Designed for real time use by care providers and managers to forecast a future ED revisit, our EMR based predictive method was prospectively validated with a reasonable level of sensitivity and specificity. After calculating the ED revisit risk scores, this information is then made available to clinicians and care-givers at the point of care to support both individual patient and population based decision-making. Moreover, high-risk patients with similar longitudinal clinical patterns can be sub-grouped for targeted post-discharge intervention in real time.

Variance analysis and two rounds of decision tree modeling process were carried out sequentially for feature selection. 152 out of 14,680 features were chosen for the final ensemble model development. Among these features, age, length of hospitalized stay, previous ED or inpatient histories, and chronic conditions were also the predictors of ED utilization found by other studies [9–11, 31]. There are 6-variable risk assessment tools that have been successfully validated and widely applied in ED settings, including Identification of Seniors At Risk (ISAR) [19, 32], Triage Risk Screening Tool (TRST) [12, 33], and Silver Code [18, 34]. However, these tools were developed for senior patients who had increasing risk of adverse outcomes post ED discharge. Our model, on the other hand, is a generalized tool targeting at statewide population at all age groups. Comparatively, we performed Silver Code and LACE index analysis [35] on our prospective cohort. Both tools however had poor performance with c-statistics of 0.61 and 0.57, respectively. Therefore, we concluded that our EMR-based model had better predicative results across statewide population in Maine.

Although our model achieved a prospective c-statistics of 0.73, which was higher than that of other risk assessment tools like Identification of Seniors At Risk (ISAR) [19, 36] and Triage Risk Screening Tool (TRST) [12, 14], our model would be of less utility if the analytical goal was set to achieve binary classification. In our study context, this ED risk scoring metric aimed to stratify patients in all-age, all-disease, and all-payor groups. The effectiveness of our risk stratification of ED revisit was supported by a “time to event” analysis (Additional file 10) on low-, intermediate-, and high-risk patient subgroups. Patients in higher risk categories returned to the ED earlier (prospective time to event analysis: *p* < 0.001) over the post discharge 6-month period.

Beyond identifying at risk ED discharges for potentially preventive services, a deeper understanding of both the unique and common attributes of various sub-groups may further facilitate overall management and the prevention of un-wanted ED utilization [10, 11, 31, 37]. Moreover, to be clinically useful, the risk stratification model should be iterative and facilitate exploration of the potential benefit (PPV) or burden (false positive rate) (business case) of managing sub-populations of high-risk patients. Accordingly, we sought to determine whether unique patterns of resource utilization or clusters of patient sub-populations existed among the considerable heterogeneity of the high-risk patient population when considered together. We demonstrated that among the high-risk group patients, their associated demographics, chronic conditions and varying patterns of resource consumption do not occur in isolation.

Our hypothesis was that the identified high-risk patients can be further divided into subgroups with unique clinical patterns. Thus, the providers and care managers would be empowered to device stratified care management plans to allow personalized care to reduce ED utilization. Cluster analysis revealed six clinically relevant subgroups among the high-risk patient population that were confirmed as durable upon prospective testing. These subgroups have unique patterns of demographics, disease severities, comorbidities and resource consumption, suggesting new opportunities to provide stratified care management among these groups. For example, cluster #4 and #6 had senior patients with co-occurring histories of the most diverse chronic conditions and linked to the highest utilization of clinical tests and prescriptions, which could be addressed through more targeted care management strategies. As shown in Table 2, Cluster 4# and 6# are two subgroups sharing similar characteristics in some chronic diseases. The average total lab test, average total radiology, and total chronic disease counts per person of Cluster 4# (514.73, 60, and 15.26) in prior 1 year however were higher than Cluster 6# (351.06, 38.77, and 12.2), which contributed to higher future 6-month ED utilization. We noted a decreased prevalence of the co-occurring chronic conditions in four other cluster groups of relatively younger adults with much less resource consumption. 24.8 % of cluster #1 subjects, who were not associated with any chronic disease history, may benefit from targeted care to keep them out of the emergency room (e.g. provision of a primary care physician or access to an outpatient clinic), although more analysis is needed to understand the risk drivers within this group. Currently, many existing care management strategies are directed toward single conditions. Our ED risk stratification model provides novel opportunities to experiment with new strategies of coordinated care targeting a combination of conditions across different age and demographic groups that we speculate may lead to greater case management efficacy. In addition, our analysis may facilitate targeted optimization in specific resource utilization for those patients with repeat healthcare visits, e.g. frequent radiographs and laboratory tests.

Our study analyzed the ED return risks with a focus on patient factors. However, it is plausible that some ED revisits can be due more to geographic ED resource accessibility factors. Therefore, we examined the geographic factors in relation to the ED revisit rates (summarized in Fig. 5). The heatmap graphic representation of the “hot” areas where high-volume of ED revisit rates correlated strongly with the local ED facility distribution, while less ED return rates were found in rural areas. Such findings were similar to that of previous study on ED use patterns of older adults [38]. Geographic analysis can help provide a comprehensive guidance to field ED care givers in regard to the patient geographic location, ED resource allocation, and targeted intervention delivery.

Senior patients are usually with higher rate of ED visits and end with poor outcomes, resulting repeated and frequent ED revisits. Our clustering analysis of high-risk patients identified 3 clusters with the majority at age 50+ (69.16 % of Cluster 2#, 75.26 % of Cluster 4#, and 94.04 % of Cluster 6#), compared with the rest 3 clusters made up by younger adults (less than 35: 52.86 % of Cluster 1#, 40.83 % of Cluster 3#, and 51.96 of Cluster 5#). Observations such as these suggest that a one size fits all approach to case management targeting the avoidance of ED return is likely insufficient as each of these sub-groups has unique characteristics demanding targeted post-discharge strategies. It requires providers and care managers to apply stratified care management plans accordingly to reduce the risk of ED revisit. For example, post-discharge plans including follow-up calls, clinic visits, and medications need to be designed more carefully for the elderly who are more vulnerable to poor outcomes and ED return. It is intriguing to speculate that our clustering analysis could be used for a more personalized or precise approach to prevention of unnecessary revisit that would be amenable to ongoing adjustment and adaptation according to ongoing success and failures to prevent revisit. Our risk assessment has been successfully deployed within the HIE and is made available on a real time basis. The operational advantage of the presented tool will allow post-discharge plans to be carefully designed. Accordingly, real time operational solutions such as that presented here is a necessary step in addressing the issue of repeated ED utilization contributed by older adults.

Our model and associated application were designed to track the evolving nature of post ED discharge risk of revisit, in a longitudinal manner, across all payers, all diseases and all age groups. With our ED risk model, tactics for modifying care management programs can be driven and measured against the analytical risk assessment derived from the HIE records, with knowledge of high risk population distribution among the chronic conditions and physical locations. After our initial success in ED risk modeling, we will, as a next step, focus to develop hypotheses on what factors determine the probability of a return ED visit (main outcome) or cost (secondary outcome). However, while HIE data represents an ideal source of community-wide/regional patient data, operational HIEs are not present in all States. The predictive model and patient clustering method can be applied to any clinical data set including the clinical EMRs directly as well as private HIEs within hospital networks.

The ED 6 month revisit model was deployed as part of the dashboard system, which is currently in production in Maine HIE. The platform allows real time risk profiling of all Maine HIE patients to support patient targeted care and population management. Applying analytical tools on EMR and HIE data, including the ED revisit risk model and the high-risk patient clustering method, will help health care providers effectively leverage their EMR to better understand ED service delivery while providing opportunities for improved healthcare delivery for the patients.

## Conclusions

We developed a risk model predictive of ED revisits within the 6 months’ period following the ED discharge. The model was prospectively validated on a statewide HIE database in Maine covering all payers, all diseases and all ages. Using the model each individual can be assigned a risk score at the time of discharge describing the probability of ED return in future 6 months, according to the individual’s clinical conditions in preceding 12 months. Applying our risk stratification algorithm on patients with various levels of ED resource usage can provide guidance of the care management, with a particular focus on those identified as ‘heavy users of ED services’ by our algorithm. Integrating this predictive tool into the HIE database close to real time provides opportunities of identifying clinical patterns of heavy ED users, leading to a deep understanding of healthcare resource utilization and population management, which can ultimately benefit the healthcare outcomes and patients’ life qualities.

## Additional files

Additional file 1:
**Data sources, management and security.** Descriptions of the sources of data used in this study, as well as its management and security requirements. (PDF 185 kb) Additional file 2:
**EMR feature used to develop the model.** A table lists the categories of features used to develop the final risk model. (PDF 87 kb) Additional file 3:
**Patient characteristics.** A table shows the patient characteristics included in the retrospective and prospective studies. (PDF 161 kb) Additional file 4:
**Modeling procedures.** Technical details of the training, calibration and blind testing procedures. (PDF 3912 kb) Additional file 5:
**Feature selection process.** Feature dimensions were reduced by variance analysis and importance analysis. 125 features were selected from the initial 14,680 features to build the final model. (PDF 110 kb) Additional file 6:
**Characterization of the discriminant features in the prospective data set.** Shrunken difference for the selected features to develop the ED risk model were graphed. Comparing the two cohorts (case/control or the low/medium/high risk), the shrunken differences of these discriminative features were much more pronounced in the low/medium/high risk cohort, demonstrating the effectiveness of these features in prospectively differentiating the targeted outcomes. (PDF 57 kb) Additional file 7:
**Unsupervised clustering procedure.** Technical details of the unsupervised clustering of high-risk patients. (PDF 107 kb) Additional file 8:
**“Time to event” analysis.** The ED revisit “time-to-event curve” showed a pattern of a rapid accrual with a stable and consistent ED visit rate thereafter. The population ED revisit curves, of patients with or without past history of ED visits, decreased significantly within 6 months from the ED discharge time, In the State of Maine, greater than 70 % ED patients with no past ED history and 80 % with past ED history revisited ED within 6 months past the index visit. Therefore, a 6-month cutoff is clinically reasonable. (PDF 183 kb) Additional file 9:
**Exploratory data analysis.** Our analysis found that both the total number and the percentage of patients with future 6-month ED visits increased as a functional of either the distinct chronic diagnoses (left panel) or the ED visit counts (right) in the prior 12 months. (PDF 116 kb) Additional file 10:
**“Time to event” graphic representation of the low, medium and high risk patients’ time to the next impending ED visit.** A graph shows the revisit rate as a function of the time period after discharge, of high-, medium- and low-risk patients. (PDF 185 kb)

## Abbreviations

CCI: Chronic Condition Indicator
ED: Emergency Department
EMR: Electronic Medical Record
HIE: Health Information Exchange
HIN: HealthInfoNet
ICD-9-CM: International Classification of Diseases, 9th Revision, Clinical Modification
LOINC: Logical Observation Identifier Names and Codes
NDC: National Drug Code
PPV: positive predictive value
ROC: receiver operating characteristics
