# Supplementary material for: Prospective stratification of patients at risk for emergency department revisit: resource utilization and population management strategy implications
Source: BMC Emerg Med. 2016 Feb 3;16:10. doi: 10.1186/s12873-016-0074-5 (PMC4739399; doi:10.1186/s12873-016-0074-5)
Supplement: Additional file 1: — Data sources, management and security. Descriptions of the sources of data used in this study, as well as its management and security requirements. (PDF 185 kb) [file 12873_2016_74_MOESM1_ESM.pdf]

Data sources, management and security.

Data used for this study were directly collected by the Maine's HIE ORION/IBM Initial/CDC database which managed real-time data transition (HL-7 message transition). The Maine HIE went live in 2009 and now contains records for close to all of Maine residents and is connected to the majority of health care facilities in Maine. There are currently 475 facilities connected to the Maine HIE including 376 physician offices, 12 behavioral health facilities, 15 critical access hospitals, 37 federally qualified health centers (FQHC), 23 hospitals, and 12 long-term care facilities. The HIE includes records for 1.35 million individuals including in-state and out-of-state residents. Over 90% of Maine residents have a record in the database. HIN is an independent, nonprofit organization operating the HIE in Maine. It maintains an opt-out consent process for general medical information and an opt-in patient consent for certain behavioral health and HIV related information as required by Maine State law. The HIE has just over a 1% patient opt-out rate.

We (HBI Solutions, Inc) managed a sequential staging data warehouse to extract, transform and load entire EMR data from HIE system. Data cleaning and integration were applied for handling error and data quality issue. And then an analysis database including data attribute described in method part was built based on the staging database for learning and prediction process.

The work was performed under a business and development arrangement between HealthInfoNet (http://www.hinfonet.org), the operators of the Maine Health Information Exchange and HBI Solutions, Inc. (HBI) located in California. HBI is a subcontractor to HealthInfoNet to develop and implement predictive risk models to be used by HealthInfoNet member providers. HealthInfoNet is responsible for security and access to its members' data and has established data service agreements (DSAs) restricting unnecessary exposure of information. All data analysis and modeling for this manuscript was performed on HealthInfoNet servers and data was accessed via secure connections controlled by HealthInfoNet. Access to the data used in the study requires secure connection to HealthInfoNet servers and should be requested directly to HealthInfoNet.
