# Supplementary material for: Prospective stratification of patients at risk for emergency department revisit: resource utilization and population management strategy implications
Source: BMC Emerg Med. 2016 Feb 3;16:10. doi: 10.1186/s12873-016-0074-5 (PMC4739399; doi:10.1186/s12873-016-0074-5)
Supplement: Additional file 2: — EMR feature used to develop the model. A table lists the categories of features used to develop the final risk model. (PDF 87 kb) [file 12873_2016_74_MOESM2_ESM.pdf]

EMR feature used to develop the model.

Feature group	Feature number	Feature description (12 month clinical history before ED discharge)
Encounter history	104	Visit counts of different encounter types (E/O/I/P/R) *
		The accumulated length of hospitalized stay
		Counts of historical chronic disease diagnoses
		Counts of total and no redundant total radiographic and laboratory tests, and outpatient prescriptions
Demographics	12	Female, male
		Income, education, payer
		Age group is defined by age at ED admission (0, 1-5yr, 6-12yr, 13-18yr, 19-34yr, 35-49yr, 50-65yr, 65+yr) **
Facility	12	Different facilities
Procedure	2	Counts for different primary procedure and secondary procedure
Chronic disease condition	1	Counts for chronic disease diseases
Diagnosis	10	Counts for primary diagnosis and secondary diagnosis
Laboratory test	2	Counts for different laboratory test results
Outpatient prescriptions	9	Counts for different outpatient prescriptions

* Encounter type descriptions: E-Emergency, O-Outpatient, I-Inpatient, P-Pre admission, R-Recurring admission, **yr-year
