# Supplementary material for: Prospective stratification of patients at risk for emergency department revisit: resource utilization and population management strategy implications
Source: BMC Emerg Med. 2016 Feb 3;16:10. doi: 10.1186/s12873-016-0074-5 (PMC4739399; doi:10.1186/s12873-016-0074-5)
Supplement: Additional file 3: — Patient characteristics. A table shows the patient characteristics included in the retrospective and prospective studies. (PDF 161 kb) [file 12873_2016_74_MOESM3_ESM.pdf]

Patient Characteristics

Characteristics	Retrospective (Jan.1, 2012 – Dec.31, 2012)		Prospective (Jan.1.2013 – Jun.30, 2013)	
	Control N = 167132	Case N = 126329	Control N = 107070	Case N = 86816
Male, %	48.02	45.54	47.72	45.11
Age (year), %				
≤ 18	20.97	14.83	21.10	14.32
19 - 65	60.76	68.68	60.78	69.10
≥ 65	18.27	16.49	18.12	16.58
Insurance status, %				
Blue Cross	2.12	1.04	2.59	1.21
Medicaid/Medicare	8.45	12.07	15.16	21.60
Commercial	3.95	2.29	6.30	3.75
Free Care/Self Pay	1.87	2.12	3.28	3.59
Others/Unknown	83.61	82.47	72.67	69.85
Median family income estimate, median (IQR)	58750 (49148, 66484)	55108 (47644, 62090)	58984 (47750, 68082)	55108 (47644, 63440)
Education, median (IQR)				
Percent high school graduate or higher	89.40 (84.40, 91.40)	89.00 (86.00, 91.40)	89.50 (86.40, 92.70)	88.80 (86.00, 91.80)
Percent bachelor's degree or higher	22.20 (16.90, 29.00)	21.70 (15.00, 26.60)	22.90 (16.20, 32.10)	22.00 (14.60, 28.30)
Health service utilization in past 12 months before ED discharge				
Percent encounters with outpatient visits	34.79	32.61	35.18	34.18
Percent encounters with ED visits	21.59	30.31	21.31	31.51
Percent encounters with inpatient visits	5.29	8.61	5.44	9.31
No. of medications, median (IQR)	0 (0,15)	6 (0,89)	2 (0,21)	9 (0,68)
No. of lab tests, median (IQR)	0 (0,46)	23 (0,100)	0 (0,52)	33 (0,133)
No. of radiology, median (IQR)	0 (0,1)	0 (0,2)	0 (0,1)	0 (0,2)
Percent encounters with chronic diseases	24.71	26.12	25.25	28.05
