# Supplementary material for: Prospective stratification of patients at risk for emergency department revisit: resource utilization and population management strategy implications
Source: BMC Emerg Med. 2016 Feb 3;16:10. doi: 10.1186/s12873-016-0074-5 (PMC4739399; doi:10.1186/s12873-016-0074-5)
Supplement: Additional file 4: — Modeling procedures. Technical details of the training, calibration and blind testing procedures. (PDF 3912 kb) [file 12873_2016_74_MOESM4_ESM.pdf]

(I) The data model

Through the exploratory study, we found that the ED visit history and chronic condition were two discriminative features related to the ED revisit (results not shown). Denote x_i as features except chronic condition and ED history, c_i as chronic condition and e_i, ED history. The data model was built through the law of total probability,

$$P(ED_i|x_i) = \sum_{c_i,e_i} P(ED_i|x_i,c_i,e_i)P(c_i,e_i)$$

As the $P(c_i, e_i)$ was known for a given encounter, we had

$$P(ED_i|x_i) = \begin{cases} P(ED_i|x_i,c_i=0,e_i=0)P(c_i=0,e_i=0) & ED\ history\ (-)\ \text{chronic}\ (-) \\ P(ED_i|x_i,c_i=0,e_i=1)P(c_i=0,e_i=1) & ED\ history\ (-)\ \text{chronic}\ (+) \\ P(ED_i|x_i,c_i=1,e_i=0)P(c_i=1,e_i=0) & ED\ history\ (+)\ \text{chronic}\ (-) \\ P(ED_i|x_i,c_i=1,e_i=1)P(c_i=1,e_i=1) & ED\ history\ (+)\ \text{chronic}\ (+) \end{cases}$$

This can be regarded as a Bayesian model [1] with a probabilistic likelihood and a deterministic prior. At the perspective of tree, the deterministic prior was like a decision node with four branches, whose decision rule was defined on the ED history and chronic condition.

The outcome probability the patient would have a ED revisit was determined as

$$P(ED_i|x_i) = P(ED_i|x_i,c_i=m,e_i=n)P(c_i=m,e_i=n)$$

where m and n were the values of ED history and chronic condition. This equation indicated that with four likelihoods, the model can forecast the result. In this paper, each likelihood was modeled using ensemble learning/model average

$$P(ED_i|x_i,c_i=m,e_i=n) = F\left(\frac{1}{\sum_b w_b}\sum_b w_b f_b(x_i)\right)$$

where w_b were weights, f_b were single models, F is a monotonic mapping function to map the outcome as a probability.

(II) The likelihood model

Denote the time t, the survival function was defined as

$$S(t|x_i) = \mathrm{P}(T > t|x_i)$$

where T indicated for the time to an ED revisit and x_i was a sample. The survival function was the probability that a patient did not have a ED revisit within t days post the discharge of the current ED visit. The cumulative hazard function was then defined as

$$H(t|x_i) = -\log S(t|x_i)$$

To predict whether a patient will have ED revisit, $H(t|x_i)$ was modeled using an ensemble learning method, called random survival forests [2, 3]. When the predicted cumulative hazard is higher at time t, the patient is more likely to have a ED revisit at that time.

The basic idea of random survival forests was the bagging and random selection. Multiple groups of bootstrapped samples were drawn from the training dataset, of which each one was employed to build a decision tree model without pruning using a subset of their features through random selection. By averaging the outcomes $\widehat{H}_b(t|x_i)$ of all decision trees, the predicted cumulative hazard function $\widehat{H}(t|x_i)$ was estimated as

$$\widehat{H}(t|x_i) = \frac{1}{ntree}\sum_{b=1}^{ntree} \widehat{H}_b(t|x_i)$$

In this paper, we set the hazard score to be the value of cumulative hazard function at time $t = 180$, which was the cumulative hazard of ED revisit at 6 months post discharge. Then the likelihood was

$$P(ED_i|x_i, c_i = m, e_i = n) = 1 - 2^{-\widehat{H}(t=180|x_i)}$$

Recall the general form of the ensemble learning based likelihood, in the random survival forests model, w_b were equally assigned, f_b was decision tree, F was shown above and was monotonic. The software we utilized for random survival forests training and predicting was randomForestSRC [4].

(III) Calibration

As the survival model output the cumulative hazard, we calibrate the model on $\widehat{H}(t = 180|x_i)$ directly. As the mapping between likelihood and hazard was monotonic, this operation would derive the same results with that from the calibration on the probability. A calibrated cohort was used to calibrate the predictive scoring threshold to create a risk measure for an individual ED post discharge sample. Applying the Step (II) model to each sample i in the cohort, the derived predictive scores $\widehat{H}(t|x_i), \mathrm{i} = 1, \dots, \mathrm{N}$ were ranked.

For each value of V, we can calculate the positive predictive value (PPV) as follows.

$$\mathrm{PPV} = f(V) = \sum_{i=1}^{N} I\big(\widehat{H}(t|x_i) - V\big)J(x_i) \,/ \sum_{i=1}^{N} I\big(\widehat{H}(t|x_i) - V\big)$$

where

$$I(x) = \begin{cases} 1 \; x > 0 \\ 0 \; other \end{cases} \qquad J(x) = \begin{cases} 1 \; x \in X_{case} \\ 0 \; x \in X_{ctrl} \end{cases}$$

and X_{case} and X_{ctrl} denote the patients who have ED revisit and never have ED visit in 6 months after discharge. In this way we have a mathematic function mapping predictive values to PPVs.

Our ED algorithm was set to segregate the ED post discharge population into subgroups with different risks of 6-month ED revisits. The risk measure is defined as $f(V)$ which is between 0 and 100. Given a risk level L, patients with measures larger than or equal to a risk level L will have the proportion of L% to have ED revisits in the next 6 months.

We obtained two thresholds V_h, V_m from this mapping.

$$f(V_h) = 70$$

$$f(V_m) = 30$$

Then we stratified the patients into three risk groups

High risk group:

$$\widehat{H}(t|x_i) \geq V_h$$

Intermediate risk group:

$$V_m \leq \widehat{H}(t|x_i) < V_h$$

Low risk group:

$$\widehat{H}(t|x_i) < V_m$$

(IV) Blind test

Blind test cohort is an independent naive sample set, which was compiled to blind test the method's performance. The aim of this step is to critically assess the utility of the risk measure before statewide prospective validation in Maine. Each blind test cohort sample's future 6-month risk measure was computed as described in Step (II). Again we applied the Step (III) model to each sample x_i in blind test cohort to derive the predictive scores $\widehat{H}(t|x_i)$, i $= 1, \ldots$,N, and calculated the AUC score for the cohort as described in Step (III) analysis. The derived predictive scores $\widehat{H}(t|x_i)$, i $= 1, \ldots,$ N were ranked, and the AUC score was computed as following:

$$\text{AUC} = \frac{1}{nm}\sum_{i=1}^{n}\sum_{j=1}^{m} I(\widehat{H}(t|x_i) > \widehat{H}(t|x_j))$$

Reference

1. Murphy KP: **Machine Learning: a probabilistic perspective**: The MIT Press; 2012.
2. Ishwaran H, Kogalur UB: **Random survival forests for R**. *R News* 2007, **7**(2):25-31.
3. Ishwaran H, Kogalur UB, Blackstone EH, M.S. L: **Random survival forests.** . *Ann Appl Statist,* 2008, **2**:841-860.
4. Ishwaran H, Kogalur UB: **Random Forests for Survival, Regression and Classification (RF-SRC)**. In.; 2015.
