# Supplementary figures and images for: Prospective stratification of patients at risk for emergency department revisit: resource utilization and population management strategy implications
Source: BMC Emerg Med. 2016 Feb 3;16:10. doi: 10.1186/s12873-016-0074-5 (PMC4739399; doi:10.1186/s12873-016-0074-5)

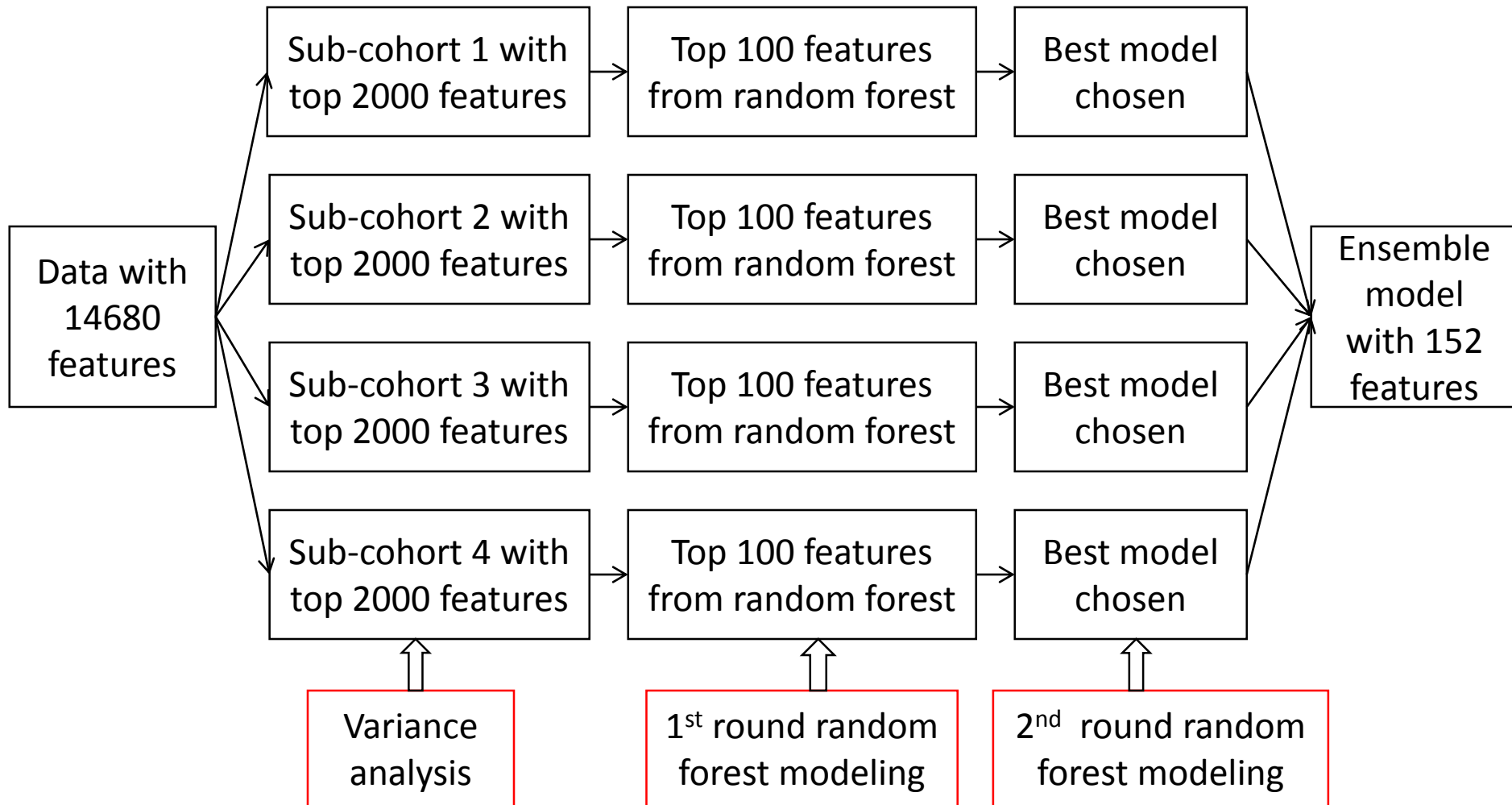

Supplement: Additional file 5: — Feature selection process. Feature dimensions were reduced by variance analysis and importance analysis. 125 features were selected from the initial 14,680 features to build the final model. (PDF 110 kb) [file 12873_2016_74_MOESM5_ESM.pdf]

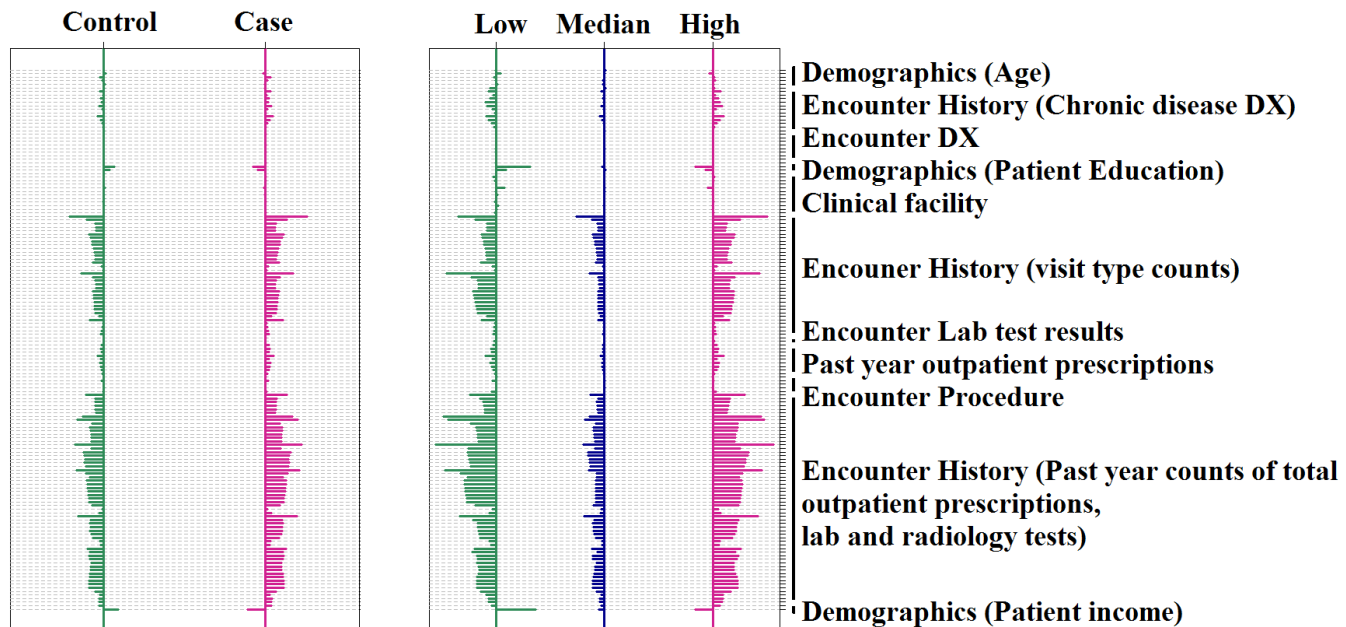

Supplement: Additional file 6: — Characterization of the discriminant features in the prospective data set. Shrunken difference for the selected features to develop the ED risk model were graphed. Comparing the two cohorts (case/control or the low/medium/high risk), the shrunken differences of these discriminative features were much more pronounced in the low/medium/high risk cohort, demonstrating the effectiveness of these features in prospectively differentiating the targeted outcomes. (PDF 57 kb) [file 12873_2016_74_MOESM6_ESM.pdf]

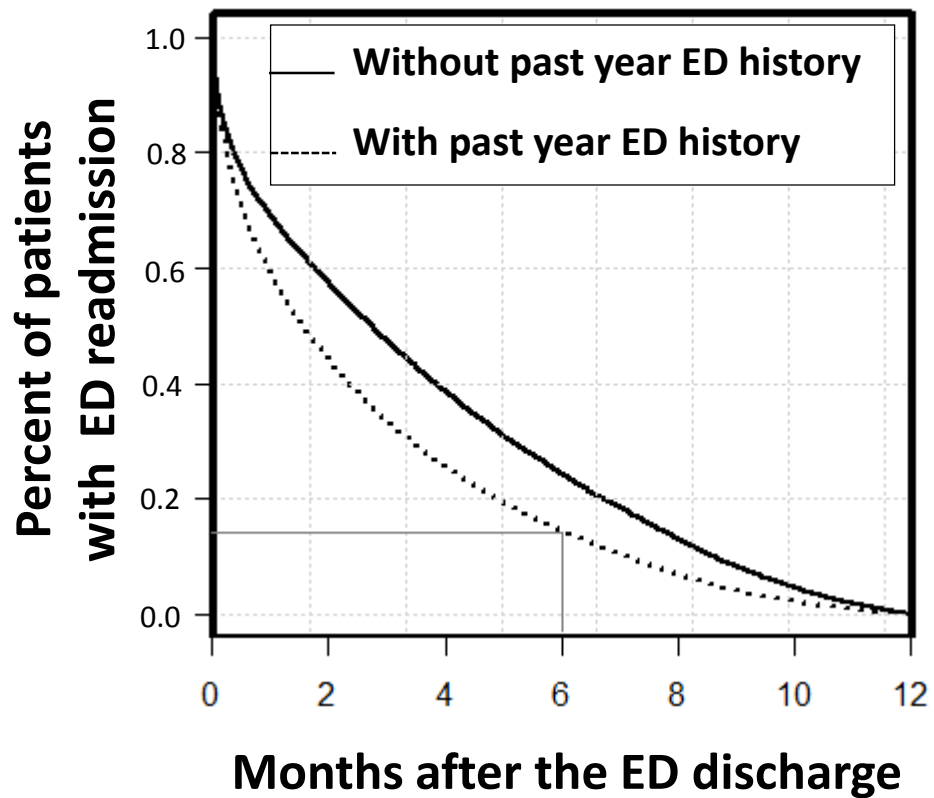

Supplement: Additional file 8: — “Time to event” analysis. The ED revisit “time-to-event curve” showed a pattern of a rapid accrual with a stable and consistent ED visit rate thereafter. The population ED revisit curves, of patients with or without past history of ED visits, decreased significantly within 6 months from the ED discharge time, In the State of Maine, greater than 70 % ED patients with no past ED history and 80 % with past ED history revisited ED within 6 months past the index visit. Therefore, a 6-month cutoff is clinically reasonable. (PDF 183 kb) [file 12873_2016_74_MOESM8_ESM.pdf]

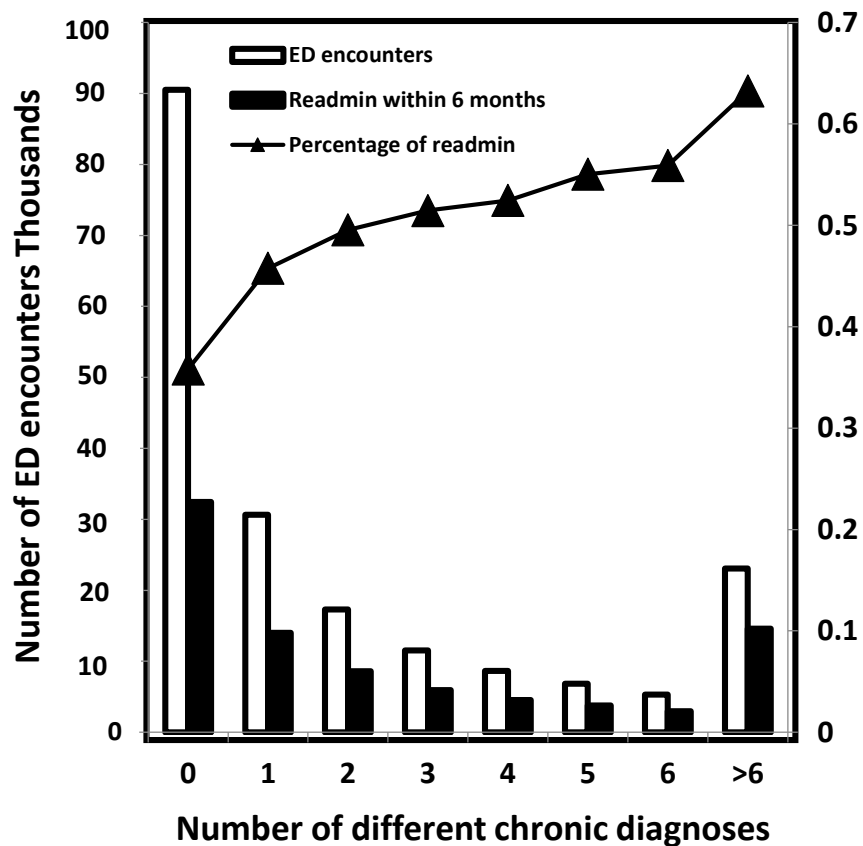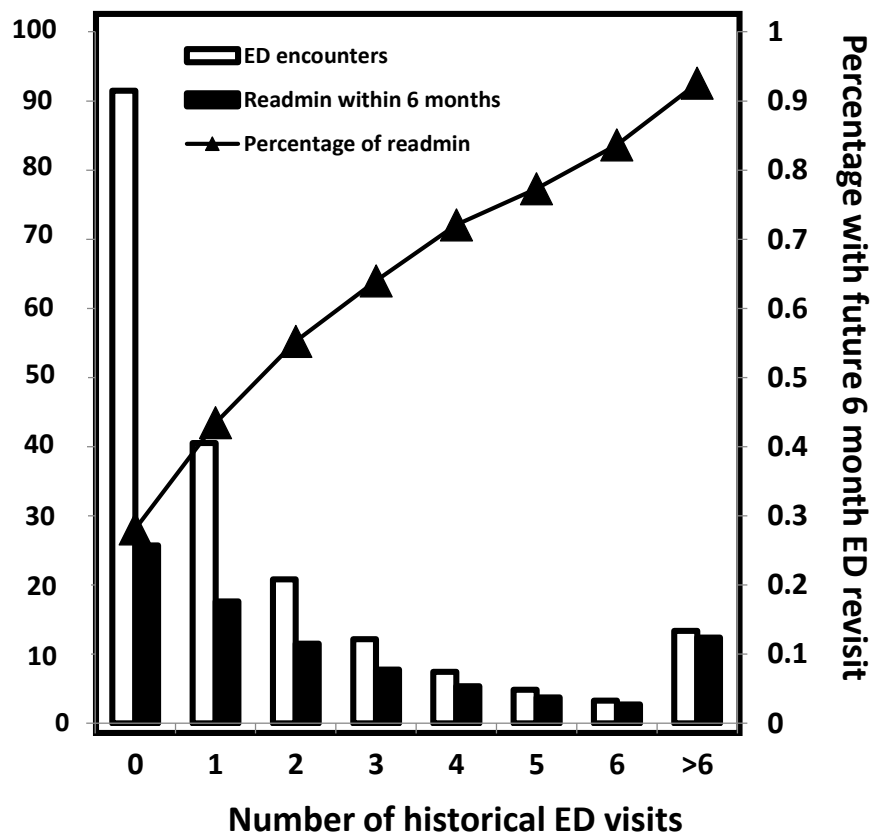

Supplement: Additional file 9: — Exploratory data analysis. Our analysis found that both the total number and the percentage of patients with future 6-month ED visits increased as a functional of either the distinct chronic diagnoses (left panel) or the ED visit counts (right) in the prior 12 months. (PDF 116 kb) [file 12873_2016_74_MOESM9_ESM.pdf]

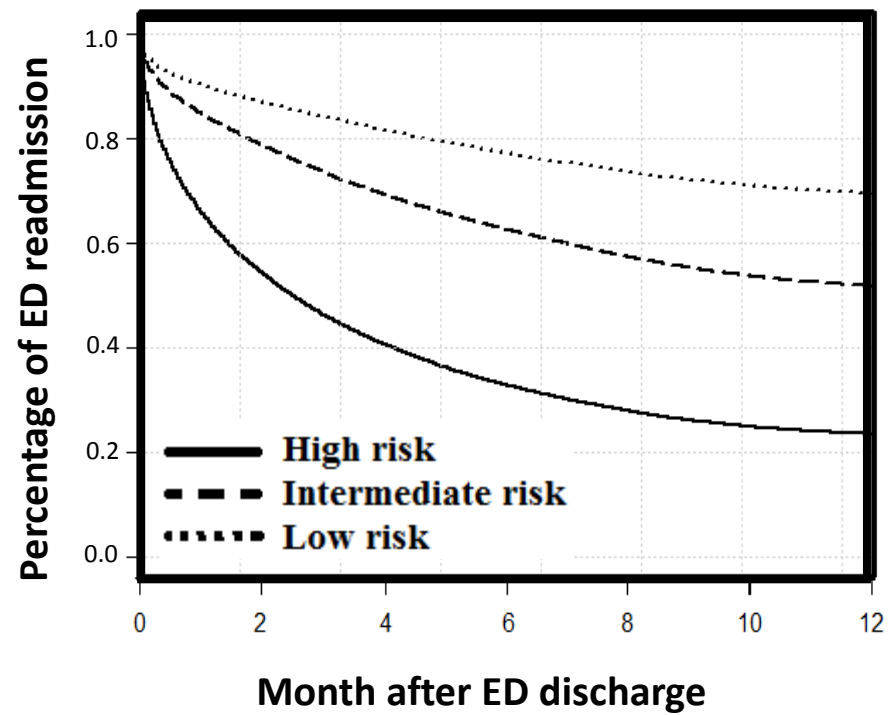

Supplement: Additional file 10: — “Time to event” graphic representation of the low, medium and high risk patients’ time to the next impending ED visit. A graph shows the revisit rate as a function of the time period after discharge, of high-, medium- and low-risk patients. (PDF 185 kb) [file 12873_2016_74_MOESM10_ESM.pdf]
