# Supplementary material for: Prospective stratification of patients at risk for emergency department revisit: resource utilization and population management strategy implications
Source: BMC Emerg Med. 2016 Feb 3;16:10. doi: 10.1186/s12873-016-0074-5 (PMC4739399; doi:10.1186/s12873-016-0074-5)
Supplement: Additional file 7: — Unsupervised clustering procedure. Technical details of the unsupervised clustering of high-risk patients. (PDF 107 kb) [file 12873_2016_74_MOESM7_ESM.pdf]

Unsupervised clustering procedure.

To reduce high dimensional EMR features for detecting cohort pattern, we used principle component analysis (PCA) to divide the high risk patients of 6-month ED return identified by our algorithm in the prospective cohort into distinctive groups, based on demographics, primary diagnosis and procedure, and chronic disease conditions. The features for high-risk patients are projected to a lower dimensional subspace with largest variances.

$$T_i^k = X_i \cdot w_k$$

Where X_i is EMR feature matrix for each high-risk patient, and w_k is the set of vectors of weights that map each patient feature vector X_i to a new vector of principal component scores T_i^k. And we computed w_1 by solving following objective functions (1) and (2) and w_k by iterating objective function (3) based on the first k-1 principal components,

$$w_1 = \arg\max_{\|W\|=1}\left\{\sum_i \left(T_i^1\right)^2\right\} = \arg\max_{\|W\|=1}\left\{\sum_i \left(X_i \cdot w\right)^2\right\}$$

$$W_1 = \arg\max\left\{\frac{w^T X^T X w}{w^T w}\right\}$$

$$w_k = \arg\max\left\{\frac{w^T (X - \sum_{n=1}^{k-1} X w_n w_n^T)^T (X - \sum_{n=1}^{k-1} X w_n w_n^T) w}{w^T w}\right\}$$

And then K-means algorithm was applied on the top of principal components T_i^k subspace of PCA to find potential patient patterns for 6-month ED return [46]. We used K=6 to implement initial k means set for the algorithm and calculate the Euclidean centroid m to generate finial clusters,

$$m_i^{t+1} = \frac{1}{|C_i^t|}\sum_{x_j \in C_i^t} x_j$$

Where C_i is the i_{th} cluster in total 6 clusters, and x represents the previous principal components T^k.

Unique patterns revealed by the clustering results were analyzed to characterize the high-risk subjects identified by our ED algorithm.
